# Targeted Transgene Expression in Cholinergic Interneurons in the Monkey Striatum Using Canine Adenovirus Serotype 2 Vectors

**DOI:** 10.3389/fnmol.2020.00076

**Published:** 2020-05-15

**Authors:** Anne-Caroline Martel, Heba Elseedy, Marina Lavigne, Jennyfer Scapula, Antoine Ghestem, Eric J. Kremer, Monique Esclapez, Paul Apicella

**Affiliations:** ^1^CNRS, Institut de Neurosciences de la Timone, Aix Marseille University, Marseille, France; ^2^INSERM, Institut de Neurosciences des Systèmes, Aix Marseille University, Marseille, France; ^3^Department of Zoology, Alexandria University, Alexandria, Egypt; ^4^CNRS, Institut de Génétique Moléculaire de Montpellier, Montpellier, France

**Keywords:** viral vector transduction, acetylcholine, microcircuit analysis, basal ganglia, nonhuman primate

## Abstract

The striatum, the main input structure of the basal ganglia, is critical for action selection and adaptive motor control. To understand the neuronal mechanisms underlying these functions, an analysis of microcircuits that compose the striatum is necessary. Among these, cholinergic interneurons (ChIs) provide intrinsic striatal innervation whose dysfunction is implicated in neuropsychiatric diseases, such as Parkinson’s disease and Tourette syndrome. The ability to experimentally manipulate the activity of ChIs is critical to gain insights into their contribution to the normal function of the striatum and the emergence of behavioral abnormalities in pathological states. In this study, we generated and tested CAV-pChAT-GFP, a replication-defective canine adenovirus type 2 (CAV-2) vector carrying the green fluorescent protein (GFP) sequence under the control of the human choline acetyltransferase (ChAT) promoter. We first tested the potential specificity of CAV-pChAT-GFP to label striatal ChIs in a rat before performing experiments on two macaque monkeys. In the vector-injected rat and monkey striatum, we found that GFP expression preferentially colocalized with ChAT-immunoreactivity throughout the striatum, including those from local circuit interneurons. CAV-2 vectors containing transgene driven by the ChAT promoter provide a powerful tool for investigating ChI contributions to circuit function and behavior in nonhuman primates.

## Introduction

Numerous studies have suggested that the striatum, the main recipient of afferents to the basal ganglia, has a critical function in motor control and motivation. This structure mostly consists of GABAergic spiny projection neurons (SPNs) that target the output nuclei of the basal ganglia. The activity of SPNs is dependent upon excitatory inputs from the cortex and thalamus, under the control of dopaminergic and cholinergic modulation and different types of GABAergic interneurons (Silberberg and Bolam, 2015). The cholinergic innervation of the striatum mainly arises from cholinergic interneurons (ChIs) scattered throughout the striatum. Although they make up a small fraction of striatal cells, ChIs exert a powerful influence on SPN activity (Calabresi et al., 2000; Pisani et al., 2007) and strongly modulate DA transmission in the striatum (Threlfell and Cragg, 2011). It has been suggested that the dysfunction of ChIs is relevant to the pathophysiology of basal ganglia. As an example, a reduction in the density of ChIs has been reported in brains from patients with Tourette syndrome (Kataoka et al., 2010), a neuropsychiatric disorder characterized by abnormal repetitive movements and altered behavioral flexibility. However, it remains unclear how these interneurons regulate the functioning of the striatal network underlying adaptive behavior. A major goal of research in this area is to develop animal models in which the activity of ChIs can be selectively modified to mimic behavioral impairments observed in human brain pathology.

The advent of tools for transgenics and genome manipulation has provided an opportunity to manipulate striatal microcircuits at an unprecedented level of accuracy. In this regard, there is a growing interest in the application of genetic tools to investigate the causal role of striatal ChIs in behavior. Transgenic rodents expressing Cre-recombinase in cholinergic neurons have allowed targeted approaches for specific manipulation of ChIs by opto- and chemogenetic approaches (Witten et al., 2010; Ztaou et al., 2016; Aoki et al., 2018). However, differences between rodent and primate brains limit the generality of insights into how striatal ChIs might contribute to adaptive behavior.

Among viral vector platforms, adeno-associated virus (AAV) and canine adenovirus type 2 (CAV-2) vectors are widely used in rodents and primates to express transgenes in neurons into various brain regions. Each platform has advantages and disadvantages. Due to the CAV-2 vector’s capacity to harbor expression cassettes of 7–30 kb (Junyent and Kremer, 2015; vs. ~4.7 kb for a typical AAV vector), and our downstream goals to use expression cassettes of greater than 5 kb, we opted for the latter.

In the present study, we developed a viral vector that uses an acetylcholine-specific promoter to selectively express a transgene in the ChI population of the primate striatum. Using CAV-pChAT-GFP, a canine adenovirus type 2 (CAV-2) vector carrying the green fluorescent protein (GFP) gene under the control of the human choline acetyltransferase promoter (pChAT), we report its ability to restrict GFP expression to striatal ChIs.

## Materials and Methods

### Animals

All experimental procedures were approved by the Institutional Animal Care and Use Committee of INT (Permission Number: 14675-2018041009396760) and complied with the rules of the European Community Council Directive (2010/63/EU) for the care and use of laboratory animals. One adult male Long Evans rat (300 g) and two adult male macaques [one cynomolgus, *Macaca fascicularis*, (monkey P), 10.7 kg and one rhesus, *Macaca mulatta*, (monkey C), 11 kg] were used in this study. Monkeys C was chronically implanted with a head-fixation device and a recording chamber placed above the striatum. In this animal, injection coordinates were chosen based on electrophysiological mapping of the striatum we performed over several weeks before injections. Typically, the dorsal border of the striatum was identified by an increase of background noise and irregular spike activity after passing the cortex and underlying white matter. Striatal neurons (mostly output neurons or SPNs) were identified by their characteristic low-frequency discharge. These are different from neighboring structures, such as globus pallidus and adjacent fiber bundles (internal capsule and anterior commissure).

### CAV-2 Vectors

**Figure 1:**
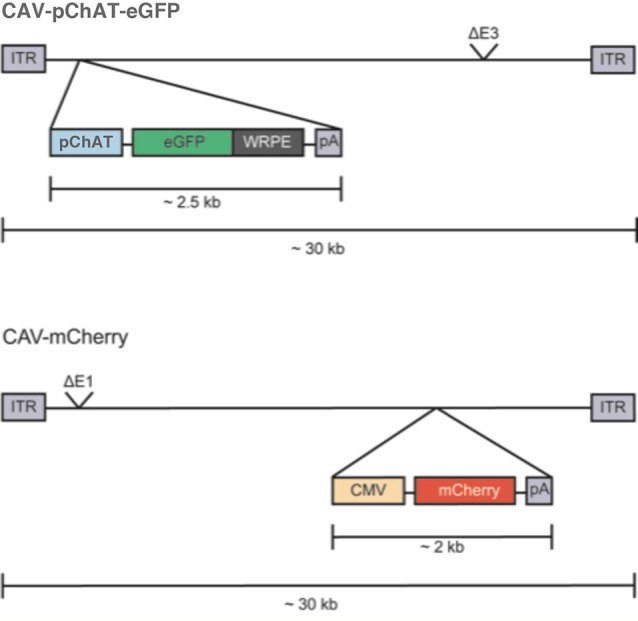
Schematic representation of the CAV-2 construct. CAV-2 vector genomes (~32 kbp) are flanked by an inverted terminal repeat (ITR) of ~200 bp. “pChAT-eGFP-WRPE-pA” is the expression cassette that replaces the E1 region in the CAV-2 genome. “pChAT” is the promoter driving expression of choline acetylase gene, GFP is the optimized open reading frame (ORF) for green fluorescent protein (GFP) from jellyfish, WRPE is a sequence from a woodchuck hepatitis virus that increases mRNA stability and protein yield when placed 3′ to an ORF, and pA is the polyadenylation signal from human growth hormone gene (GH1). The CAV-2 E3 region is also deleted (ΔE3) to increase the packaging capacity.

CAV-pChAT-GFP and CAV-mCherry are E1/E3-deleted replication-defective vectors. The constructs used in this study are schematically shown in Figure 1. For CAV-pChAT-GFP, the cassette contains the 513 bp ChAT promoter, provided by Dr. W. Stauffer (University of Pittsburgh, PA, USA) and amplified with primers 5′-gaatgcaattgcgcgatttatt taaatcccgggagcagggggtg-3′ and 5′-tcgcccttgctcaccatggcggccgc ggtggcgagccaggcc-3′), upstream of the GFP open reading frame (ORF; amplified with primers 5′-ggcctggctcgccaccgcg gccgccatggtgagcaaggg-3′ and 5′-cagaggttgattgaattcttact tgtacagctcgtc-3′) from pEF-GFP; a gift from Connie Cepko (Addgene plasmid #11154), followed by the woodchuck hepatitis virus posttranscriptional regulatory element (WPRE), and polyadenylation sequences (amplified with primers 5′-gacgagctgtacaagtaagaattc aatcaacctctg and 5′-aggtacccgccgcgcgatttatttaaa taaggacagggaagggagc from the plasmid of Dr. W. Stauffer). The pChAT-GFP-polyA cassette was cloned in the E1 region of the CAV-2 vector by SLiCE and produced as described (Del Rio et al., 2019). CAV-mCherry contains the cytomegalovirus (CMV) early promoter, the mCherry ORF, and a polyA signal, cloned in the CAV-2 E3 region (Figure 1).

### Surgery for Viral Vector Injections

The rat was anesthetized by an intraperitoneal injection of ketamine (100 mg/kg)/xylazine (10 mg/kg) solution and prepared for stereotaxic injections of the viral vectors. CAV-mCherry (10^9^ pp/μl) and CAV-pChAT-GFP (10^9^ pp/μl) were pressure-injected into the dorsal striatum of respectively the left and right hemispheres. Two injections (0.5 μl each for CAV-mCherry and 1 μl each for CAV-pChAT-GFP) were performed for each vector. After surgery, the skin was sutured, prophylactic antibiotics were injected (Baytril, 5 mg/kg i.m.), and the animal was replaced in its home cage for a survival period of 7 days.

Both monkeys received injections of CAV-2 vectors into the putamen (same constructs as those used in the rat). Monkey P had injections in both sides of the brain and monkey C on one side (i.e., the hemisphere where the recording chamber was placed). Under general anesthesia (sevoflurane 2%) and aseptic conditions, the head of monkey P was fixed in a stereotaxic frame and small holes were drilled into the skull at chosen coordinates above the striatum. After puncturing the dura, a 30-gauge needle connected to a 50 μl Hamilton syringe was slowly lowered through drilled holes to the target depth. We injected a single volume of 50 μl of CAV-pChAT-GFP vector (2 × 10^9^ pp/μl, 5 μl/min) delivered in one hemisphere and 20 μl of CAV-mCherry (2 × 10^9^ pp/μl) delivered into 4 sites (5 μl per site, 2 μl/min) in the other hemisphere with tracks into the putamen at two rostrocaudal levels and two different depths per track. The distances between the injection sites were 7 mm along the rostrocaudal direction and 6 mm in depth. After each injection, the needle was left in place an additional 5 min to minimize reflux along the injection tract. When the injections were completed, the holes were filled with Spongel and the skin was sutured.

Monkey C was injected unilaterally into the putamen using a microdrive fixed on the chamber and we replaced the recording electrode with a 30-gauge needle connected to a 50 μl Hamilton syringe. The needle was positioned inside a metal guide tube designed to penetrate the dura. A total of 65 μl of the CAV-pChAT-GFP vector (2 × 10^9^ pp/μl) was injected into the putamen at two rostrocaudally different levels: one single 50 μl injection (5 μl/min) and 15 μl divided in three 5 μl boluses (2 μl/min) along the same track. Also, 15 μl of CAV-mCherry (2 × 10^9^ pp/μl) divided into three 5 μl injections (2 μl/min) separated by 2 mm along the same track were delivered at a more anterior level. After injections, the chamber was sealed with a cap. The two monkeys were monitored during recovery from anesthesia and then returned to their home cages for a survival period of 10 days.

### Histology and Immunohistochemistry

Animals were deeply anesthetized with an overdose of pentobarbital and transcardially perfused with a solution of 4% paraformaldehyde (PFA) prepared in 0.12 M sodium phosphate buffer, pH 7.4 (PB). After perfusion, blocs of the brain containing the striatum were post-fixed and processed for sectioning (coronal sections 40 μm). One out of every ten sections was stained with cresyl violet to localize the injection sites within the striatum and to determine the general histological characteristics of the tissue throughout the rostrocaudal extent of the striatum.

Selected sections at the level of the striatum were processed for simultaneous immunohistofluorescence detection of GFP, mCherry, and ChAT according to a previously described protocol (Soussi et al., 2015). Sections were incubated overnight at RT in a solution containing the following primary antibodies: mouse anti-GFP (1:100, Invitrogen), rabbit anti-RFP (1:2,000) and goat anti-ChAT (1:100, Millipore). They were incubated for 2 h in the following secondary antibodies: Alexa488-conjugated donkey anti-rabbit IgG (1:200; Invitrogen), Cy5-conjugated donkey anti-goat (1:100; Jackson ImmunoResearch Laboratories, Inc., West Grove, PA, USA), and Cy3-conjugated donkey anti-mouse (1:100; Jackson ImmunoResearch Laboratories, Inc., West Grove, PA, USA). All sections were then mounted on super frost-coated slides (Menzel GmbH and Co KG, Germany) for rat or (HistoBond+ Supa Mega, Marienfeld, Germany) for monkey, dried overnight at RT and coverslipped with Fluoromount. The specimens were analyzed with a confocal microscope (Zeiss, LSM 510).

#### Quantification of Co-localizing GFP or mCherry and ChAT

Quantitative analysis of double-labeled neurons for ChAT and GFP or mCherry was conducted to evaluate the extent of GFP expression in cholinergic neurons in the striatum. This analysis was performed in the rat and monkey P injected with CAV-pChAT-GFP and CAV-mCherry. The numbers of single- and double-labeled neurons were determined in the striatum for each animal, from five sections (400 μm apart from each other) surrounding the injection sites previously localized on cresyl violet staining adjacent sections. For each section, an image of the entire striatal region was obtained from a single confocal slice with 20× objective and sequential acquisition of the different wavelength channels (LSM 510 Zen, Zeiss). The analysis was then performed with Neurolucida software (version 7, mbfBioscience) as followed: for each confocal image, all GFP-labeled neurons were identified on the green channels and examined for co-localization with ChAT in the blue channels and/or mCherry in the red one. Then, the relative percentages of double-labeled neurons for GFP and ChAT, and mCherry and ChAT, were determined. A total of 76 GFP-labeled and 20 mCherry-labeled neurons were counted in the rat. In monkey P, 86 GFP-labeled and 30 mCherry-labeled neurons were counted.

## Results

### The Cholinergic Phenotype of Transduced Neurons After CAV-2 Injections in the Rat Striatum

**Figure 2:**
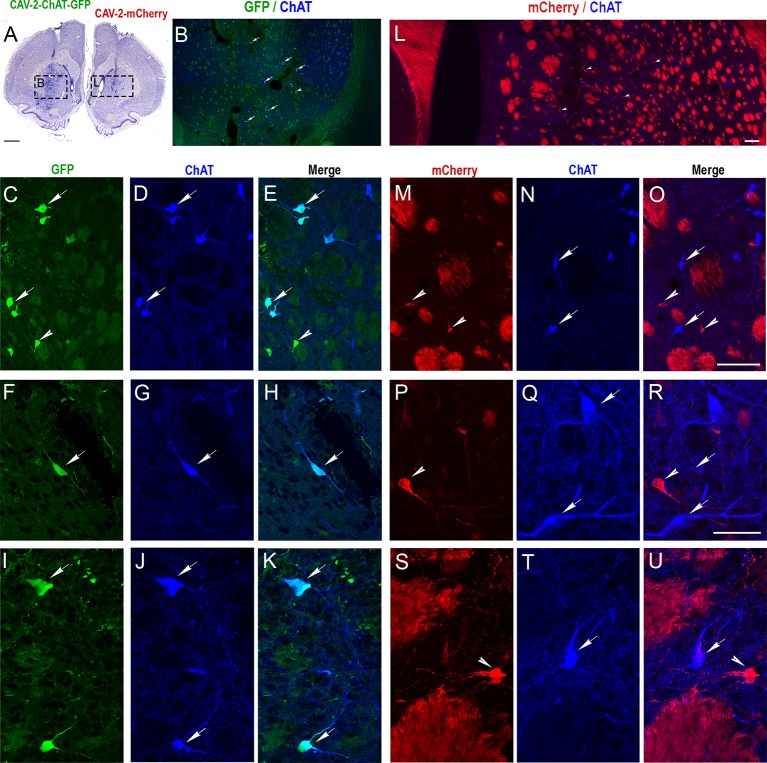
Selective expression of GFP in cholinergic interneurons (ChIs) of the rat striatum with CAV-2 containing the human ChAT promoter (CAV-pChAT-GFP). **(A)** Coronal section of the rat brain stained with Cresyl violet showing the injection sites of CAV-pChAT-GFP and CAV-mCherry in the right and left striatum respectively. **(B–U)** Immunofluorescence labeling for simultaneous detection of ChAT (blue), GFP (green) and mCherry (red) in a coronal section. Images of the fluorophores obtained by sequential acquisition of separate wavelength channels from a single confocal slice. **(B,L)** Distributions of ChAT-containing neurons as well as GFP- (**B**, green) and mCherry- (**L**, red) expressing neurons within the right and left striatum. **(C–K)** Demonstrated that many cell bodies that were GFP+ (green, arrows) contained ChAT (blue arrows). A few GFP+ (green arrowhead) did not show a detectable level of ChAT. **(E)** Merge of **(C,D)**. **(H)** Merge of **(F,G)**. **(K)** Merge of **(I,J)**. **(M–U)** Demonstrated that most mCherry+ neurons (red, arrowheads) were not labeled for ChAT. These neurons were distributed among many ChAT-containing neurons (Blue, arrows). **(O)** Merge of **(M,N)**. **(R)** Merge of **(P,Q)**. **(U)** Merge of **(S,T)**. Scale bars: **(A)**, 500 μm; **(B–D,E,L,M–O)**, 100 μm; **(F–K,P–U)**, 50 μm.

Coronal sections of the rat brain injected with CAV-pChAT-GFP and CAV-mCherry into the striatum of the right and left hemispheres, respectively (Figure 2A), were processed for simultaneous detection of GFP, mCherry, and ChAT to determine the specificity of CAV-pChAT-GFP to label cholinergic neurons of the striatum, as compared to CAV-mCherry that potentially targets all neurons.

The right striatum injected with CAV-pChAT-GFP displayed many GFP-labeled neurons. These neurons were distributed at a distance of up to 600 μm surrounding the injection site. Qualitatively, it appeared that transduced cells were more rarely observed near the injection site (Figure 2B arrows). As shown in Figures 2C,F,I, these GFP-labeled neurons displayed a large cell body with bipolar (Figure 2F) or multipolar dendrites (Figure 2I). Labeled axonal processes and en passant boutons were also observed. These neurons exhibited the size and morphology characteristics of the “classical” giant neurons presumed to be ChIs (Bolam et al., 1984; Phelps et al., 1985). While a large majority of the GFP-expressing neurons were immunolabeled for ChAT (Figures 2C–K, arrows), some GFP-expressing neurons did not contain a detectable level of ChAT (Figures 2C–E, arrowheads). Quantitative analysis performed in five sections showed that 82% of the GFP-expressing neurons were ChAT immunolabeled.

In the left striatum, many mCherry-labeled cells were observed close to the injection site and within 600 μm (Figure 2L). mCherry was distributed throughout the cell body, dendrites (Figures 2M,P,S arrowheads) and axonal processes (Figures 2P,S). As illustrated in Figures 2M–U, most of these neurons expressing mCherry (red arrowhead) were not labeled for ChAT (blue arrows). Quantitative analysis performed from 5 sections showed that only 5% of the neurons expressing mCherry were ChAT immunolabeled. This encouraging proof-of-concept pilot assay in the rat striatum provided the impetus to test CAV-pChAT-GFP in the monkey brain.

### The Cholinergic Phenotype of Transduced Neurons After CAV-2 Injections in the Macaque Striatum

**Figure 3:**
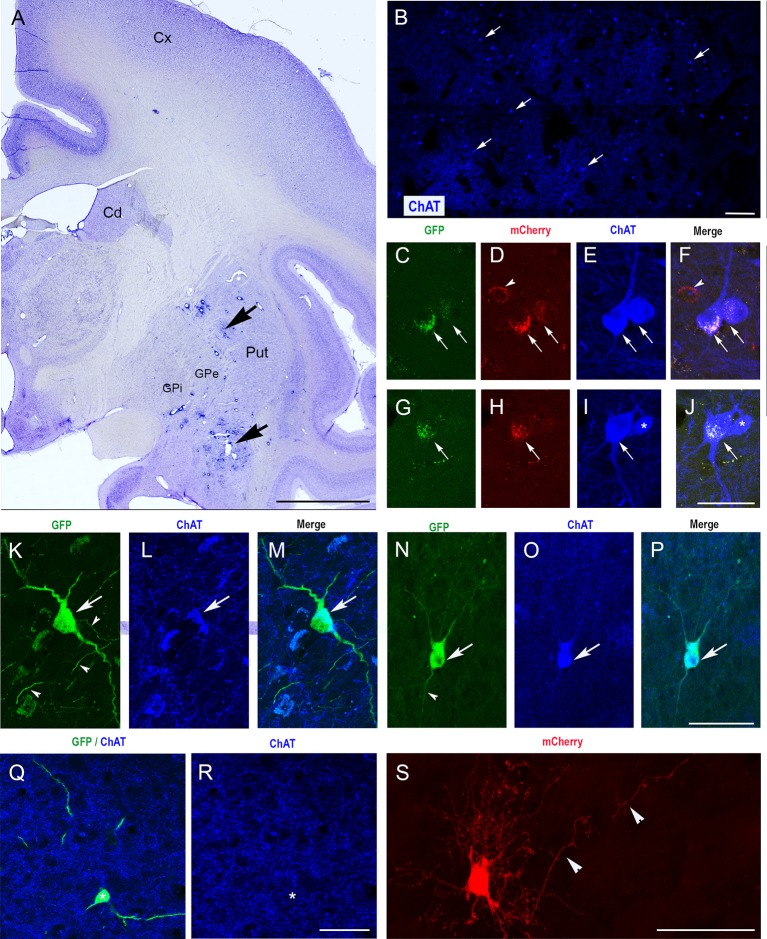
Selective expression of GFP in ChIs of the monkey striatum with CAV-2 containing the human ChAT promoter (CAV-pChAT-GFP). **(A)** Coronal section of a macaque brain stained with Cresyl violet showing the injection sites of CAV-pChAT-GFP in the striatum. **(C–S)** Immunofluorescence labeling for simultaneous detection of, GFP (green), mCherry (red) and ChAT (blue) in an adjacent coronal section. Images of the fluorophores obtained by sequential acquisition of separate wavelength channels from a single confocal slice.** (B)** Distributions of ChAT-containing neurons within the striatum. **(C–J)** In monkey C in which both CAV-2 vectors were injected in the same hemisphere, GFP and mCherry expression were restricted to the cell bodies. Most of these neurons that were labeled for both GFP (green, arrows) and mCherry (red, arrows) contained ChAT (blue) whereas neurons expressing mCherry only (red, arrowheads) were not labeled for ChAT. **(K–S)** In Monkey P in which CAV-pChAT-GFP and CAV-mCherry were injected in the right and left striatum, respectively most neurons expressing GFP (**K,N,Q**, green) or mCherry **(S)** displayed well-labeled cell bodies as well as dendritic and axonal (arrowheads) processes. **(K–P)** Most neurons expressing GFP (green, arrows) contained ChAT (Blue, arrows). **(Q,R)** A few neurons expressing GFP did not show detectable levels of ChAT. **(F)** Merge of **(C–E)**. **(J)** Merge of **(G–I)**. **(M)** Merge of **(K,L)**. **(P)** Merge of **(N,O)**. Scale bars: **(A)**, 5 mm; **(B)**, 600 μm; **(C–S)** 50 μm.

We then injected CAV-pChAT-GFP and CAV-mCherry into the striatum of two macaques. In monkey C, the two viral vectors were injected in the same hemisphere. In this monkey, only the injection sites at the posterior levels (i.e., postcommissural putamen) were properly located in the striatum (Figure 3A). Sections processed at these levels for simultaneous detection of GFP, mCherry, and ChAT showed many ChAT-containing neurons distributed through the striatum (Figure 3B) as well as GFP-labeled and mCherry-labeled neurons in the striatum. Whereas almost all the GFP-expressing neurons were labeled for ChAT and mCherry (Figures 3C–J arrows), neurons expressing mCherry only were not labeled for ChAT (Figures 3C–F arrowhead). This reflects a strong but not exclusive bias toward cholinergic neurons in a co-injection procedure. Moreover, in monkey C, expression of GFP or mCherry was limited to cell bodies, no dendritic and axonal processes were observed. The absence of “Golgi-like” labeling of GFP or mCherry expressing neurons is in contrast to that found in the rat, and could reflect toxicity due to GFP, mCherry, and/or the dose of vector during the co-injections.

To address this possibility, monkey P received injections of CAV-pChAT-GFP and CAV-mCherry in the right and left hemispheres, respectively. As in the rat striatum, the right striatum contained neurons expressing GFP with a large cell body and several dendrites with multipolar or bipolar orientation (Figures 3K,N,Q). These morphological features are consistent with those of the giant neurons (DiFiglia, 1987; Yelnik et al., 1993) that correspond to ChIs in the primate striatum. Axonal processes and en passant boutons labeled for GFP were also observed (Figures 3K,N, arrowheads). A large majority of these GFP-expressing neurons were ChAT immunoreactive (Figures 3K–P, arrows, 3Q,R). Quantitative analysis performed in monkey P on five brain sections showed that 86% of the GFP-expressing neurons were labeled for ChAT. In the striatum of the left hemisphere, neurons expressing mCherry were observed around the injection site. All these neurons displayed well-labeled cell body, dendritic and axonal processes (Figure 3S), and only 20% of these mCherry-labeled neurons co-expressed ChAT (*n* = 5 sections).

## Discussion

In the present experiment, we developed a cell-type-specific gene expression approach using a CAV-2 vector carrying a ChAT promoter to investigate the role of ChIs in nonhuman primates. The results of this pilot study indicate that CAV-2 vectors containing a human ChAT promoter can be a valuable tool to preferentially target transgene expression to the ChI population of the striatum in monkeys. This represents a promising outcome, particularly for primate models where genetic resources are underdeveloped.

### CAV-2 Vectors for Delivering Genetic Material to the Primate Brain

Previous studies have shown that CAV-2 is an effective gene transfer tool in both rodents and primates (Junyent and Kremer, 2015; Mestre-Francés et al., 2018; Lasbleiz et al., 2019). CAV-2 vectors enter axon terminals at the site of injection and are transported along the axon back to the soma, where the transgene is expressed. To our knowledge, the present study is the first that investigated the transduction properties of CAV-2 under the control of a ChAT promoter. Consistent with specific GFP expression in ChIs, we found a substantial overlap between GFP- and ChAT–immunolabeled neurons in monkey striatum, indicating that transgene expression was largely restricted to ChIs. These results demonstrate the utility of the CAV-2 vector for investigating the contributions of ChIs to striatal function and behavior, and they indicate that the ChAT promoter presently used can confer a degree of cell-type specificity to direct gene expression in the striatum of monkey.

Although the ChI system is a major contributor to the striatal cholinergic innervation, an extrinsic source of acetylcholine within the striatum also arises from the pedunculopontine and laterodorsal tegmental nuclei in the brainstem (Dautan et al., 2014). Considering that CAV-2 vectors enter axon terminals at the injection site and are transported along the axon back to the cell body, additional work will be needed to establish whether transgene expression under the control of the ChAT promoter is also present in midbrain nuclei.

### Cell-Type Targeting With the Use of Promoters

The development of gene transfer strategies with viral vectors in nonhuman primates has grown rapidly in recent years (Galvan et al., 2017). However, targeting specific types of neurons with these tools remains challenging. There are very few reports in monkeys of viral vectors that drive gene expression exclusively in a targeted neuron type with the use of promoters. Notably, a tyrosine hydroxylase promoter has been used for targeting noradrenergic neurons of the locus coeruleus (Lerchner et al., 2014) and dopaminergic neurons of the substantia nigra in macaques (Stauffer et al., 2016). Also, El-Shamayleh et al. (2017) have successfully achieved targeted transduction on cerebellar neurons in macaques using a fragment of the Purkinje cell-specific L7 promoter. These results show that cell-type-specific promoters can be used in viral vectors for targeted manipulations of specific neuronal circuits in nonhuman primates. Our results indicate that striatal delivery of CAV-2 vectors carrying a ChAT promoter sequence can confer a sufficient degree of cell-type specificity to transduce ChIs in monkey striatum. These data are comparable to the CAV-2-mediated preferential expression of a transgene in noradrenergic neurons using PRS, a catecholamine-selective synthetic promoter (Hwang et al., 2001; Li et al., 2016; Hirschberg et al., 2017).

### Technical Considerations

To increase the applicability of this methodology, further improvements in vector delivery are needed. Although we found that CAV-2 vectors preferentially transduced ChIs around the site of injection, the extent of the spread of the 90 nm CAV-2 capsid needs to be optimized because the sparse population of ChIs is scattered across large volumes of the macaque striatum. Increasing the number of coordinates where the vector is delivered and the infusion volume with Convection Enhanced Delivery (Bankiewicz et al., 2000) could be used to increase the number of ChIs expressing the transgene. Accordingly, work is now in progress to maximize the spread of viral vector and transgene expression in large regions of the macaque striatum. Also, based on ChAT immunohistochemical detection, the 513 bp ChAT promoter did not confer exclusive cell-type specificity. Replication-defective CAV-2 vectors deleted in the E1 and E3 regions have a cloning capacity of ~6 kbp, and therefore depending on the size of the transgene larger promoters/enhancer combinations could be readily incorporated. Finally, there is also uncertainty about the duration of transgene expression in longer-lasting experiments. While our work does not address long-term transgene expression in ChIs, helper-dependent CAV-2 (which have a 30 kb cloning capacity and are deleted in all CAV-2 coding regions) have led to long-term transgene expression in nonhuman primates (Mestre-Francés et al., 2018).

In conclusion, our pilot study demonstrates that the delivery of CAV-2 vectors containing a transgene driven by a ChAT promoter to the striatum results in preferential expression in ChIs in the macaque monkey. It, therefore, appears that our approach is efficient in enabling precise, targeted manipulation of a local circuit component in the striatal network of the primate. Given the complexity of the striatal circuitry, the manipulation of a single cell type that regulates striatal functions is an important step forward in mapping the local microcircuitry dysregulated in pathological conditions. The advent of molecular genetic techniques to label and manipulate specific populations of neurons opens up opportunities for linking ChI function and behavior. Our findings indicate promising capabilities of CAV-2 to enable analysis of specific microcircuits in the striatum when being combined with optogenetic or chemogenetic manipulation studies.

## Data Availability Statement

The datasets generated for this study are available on request to the corresponding author.

## Ethics Statement

The animal study was reviewed and approved by Institutional Animal care and Use Committee of INT (Permission Number: 14675-2018041009396760).

## Author Contributions

ME, PA, and EK conceived and designed experiments. ML and EK produced the virus vectors. A-CM, HE, JS, AG, ME, and PA performed experiments. ME and A-CM analyzed data and made figures. PA, ME, and EK wrote the manuscript.

## Conflict of Interest

The authors declare that the research was conducted in the absence of any commercial or financial relationships that could be construed as a potential conflict of interest.
